# In Situ Growth of Graphene on Polyimide for High-Responsivity Flexible PbS–Graphene Photodetectors

**DOI:** 10.3390/nano13081339

**Published:** 2023-04-12

**Authors:** Liangchen Hu, Jun Deng, Yiyang Xie, Fengsong Qian, Yibo Dong, Chen Xu

**Affiliations:** 1 Key Laboratory of Optoelectronics Technology, Beijing University of Technology, Ministry of Education, Beijing 100124, China; 2 Institute of Photonic Chips, University of Shanghai for Science and Technology, Shanghai 200093, China

**Keywords:** transfer-free, graphene, flexible substrate, low temperature growth

## Abstract

Graphene is an ideal material for flexible optoelectronic devices due to its excellent electrical and optical properties. However, the extremely high growth temperature of graphene has greatly limited the direct fabrication of graphene-based devices on flexible substrates. Here, we have realized in situ growth of graphene on a flexible polyimide substrate. Based on the multi-temperature-zone chemical vapor deposition cooperated with bonding a Cu-foil catalyst onto the substrate, the growth temperature of graphene was controlled at only 300 °C, enabling the structural stability of polyimide during growth. Thus, large-area high-quality monolayer graphene film was successfully in situ grown on polyimide. Furthermore, a PbS–graphene flexible photodetector was fabricated using the graphene. The responsivity of the device reached 10^5^ A/W with 792 nm laser illumination. The in-situ growth ensures good contact between graphene and substrate; therefore, the device performance can remain stable after multiple bending. Our results provide a highly reliable and mass-producible path for graphene-based flexible devices.

## 1. Introduction

With the development of modern communication technology and the internet of things, devices with high foldability and flexural resistance are increasingly developed. Flexible devices can be bent, folded and even stretched, and their applications in electronic skin, smart textiles, electronic eyes and flexible cameras are gaining wide attention [1,2,3]. Graphene, as a monolayer flexible material with high mechanical strength, has played an important role in the field of flexible electronics and optoelectronics because of its ultra-high carrier mobility, zero-band gap and superior thermal properties [4,5,6,7,8]. However, due to the rigorous growth temperature of chemical vapor deposition (CVD) graphene, transferring graphene onto a flexible substrate is the only way to fabricate graphene-based flexible devices, resulting in huge problems in the device’s long-term reliability and mass production.

Growing graphene directly on a flexible substrate is a perfect solution to the above problems, but the growth temperature needs to be lowered than the maximum withstand temperature of the flexible substrate. Now, researchers have developed various methods to synthesize graphene films at low temperatures, including using new liquid–gas carbon sources with low pyrolysis energy [9,10,11] and plasma-assisted chemical vapor deposition (PECVD) [12,13,14], gaseous aromatic hydrocarbons such as benzene and their derivatives are regarded as suitable carbon sources [15,16]. However, the continuity of the graphene is poor and cannot be used for mass device fabrication. Moreover, this compound has biological toxicity and is not environmentally friendly. For PECVD, although it can reduce the growth temperature, it still generally requires a growth temperature above 600 °C, which is unacceptable for flexible substrates. In 2021, Han et al. reported a two-step plasma-assisted thermochemical vapor deposition [17], which can directly grow high-quality graphene at 100 °C. However, the TiO_2_-x catalyst layer used in growth cannot be removed, which limits the use in flexible devices. At present, direct growth of graphene on flexible substrates for device application has not been reported.

In this paper, we realized the in situ growth of graphene on a flexible polyimide (PI) substrate. Based on the multi-temperature-zone CVD system cooperated with Cu sacrificial layer, the large-area and high-quality monolayer graphene film was successfully obtained at 300 °C. On this basis, a PbS–graphene flexible photodetector was fabricated. The PbS quantum dots (QDs) were spin-coated on the graphene to form a heterojunction. The PbS QDs as light absorption layer greatly enhance the device responsivity. Experiment results show that, under 792 nm laser irradiation, the responsivity of the device reaches 10^5^ A/W. The in situ grown graphene has a good contact with the substrate, thus ensuring the long-term stability of the device in bending. In the experiment, the photocurrent of the device remained unchanged after being bent 100 times. Our results provide a highly reliable and mass-producible path for graphene-based flexible devices.

## 2. Result and Discussion

**Figure 1:**
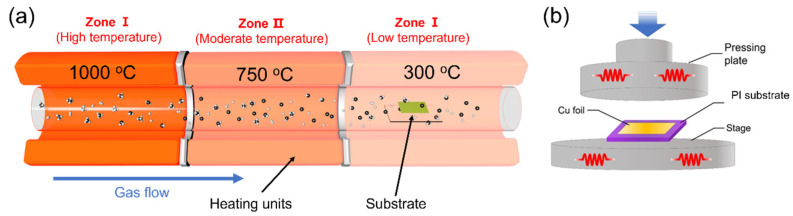
(**a**) Schematic diagram of tube furnace with gradient descent temperatures. (**b**) Schematic of bonding a single-crystal Cu sacrificial layer on the PI substrate.

As shown in Figure 1a, the multi-temperature-zone CVD system is a quartz tube furnace with three independent heated zones. In the experiment, when methane molecules passed through the high-temperature zone (zone I = 1000 °C), the C-H bonds were broken to form carbon atoms, which carried high energy and continued to flow to the low-temperature zone (zone III = 300 °C) and were adsorbed on the surface of a single-crystal Cu sacrificial layer for the growth of graphene. It should be noted that, due to the thermal radiation effect of the quartz tube, when the temperature gap between adjacent zones is large, the temperature in the low-temperature zone will not be effectively controlled. Therefore, zone II = 750 °C was added between Zone I and zone III to weaken the effect of heat conduction. By this method, monolayer graphene can be grown at 300 °C on the Cu sacrificial layer. To achieve in situ growth, the Cu sacrificial layer was pre-bonded on the PI surface (Figure 1b). After the graphene growth, we removed the Cu by a penetrating etching technique [18]. It should be noted that before the Cu layer was bonded, we pre-prepared the device electrodes on the PI substrate, therefore avoiding the damage to graphene in the device fabrication process (Methods).

**Figure 2:**
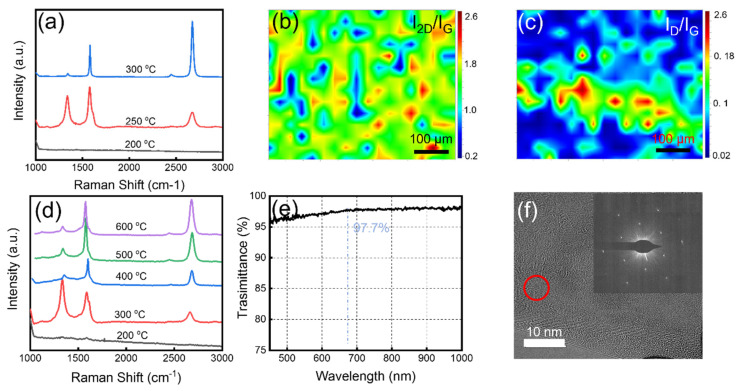
(**a**) Raman spectra of graphene grown at 200 °C, 250 °C and 300 °C. (**b**,**c**) Raman mapping images of *I*_2*D*_/*I_G_* and *I_D_*/*I_G_* of the graphene film. The measurement area is 500 μm × 500 μm. (**d**) Raman spectra of graphene grown at different temperatures without gradient temperature. (**e**) The transmittance of the obtained graphene film. (**f**) TEM image and corresponding SAED pattern of the graphene film.

The graphene quality was optimized based on Raman characterization (λ = 532 nm). Due to the weak graphene signal (Supplementary Figure S1) on PI surface, the graphene growth on the Cu foil sacrificial layer was transferred to a SiO_2_/Si substrate. Figure 2a shows the Raman spectra of graphene grown at different temperatures of Zone III. The Raman shifts for D, G, and 2D peaks of the graphene are located at 1351 cm^−1^, 1586 cm^−1^, and 2661 cm^−1^, respectively. The D peak represents the defects and disorder of the lattice in graphene [19]. In high-quality graphite or graphene, the D peak is generally very weak. The G peak is generally located at 1587 cm^−1^ and represents the in-plane vibration pattern of sp^2^ hybrid carbon atoms. The position of the G peak is very sensitive to the number of graphene layers. In general, it can be calculated by the following equation [20]:

*ω_G_* = 1581.6 + 11/(1 + *n*^1.6^)

where *ω_G_* is the peak position and n is the number of graphene layers. Bringing the measured G peak into the above equation, we can deduce that the obtained graphene is a monolayer. The 2D peak represents the vibration mode of the two photonic lattices and is secondary to the D peak. For monolayer graphene, the 2D peak is a single symmetrical peak with a half-height width of about 30 cm^−1^. As the number of layers increases, the symmetry decreases and the 2D peak splits into multiple overlapping peaks. Because the doping and stress of graphene can change the position of the G peak and the 2D peak, we also use the intensity ratio of the 2D peak and the G peak to characterize the number of graphene layers. From Figure 2a, we can see that when the growth temperature is 300 °C, the D peak almost disappears, and the peak ratio of *I*_2*D*_/*I_G_* is close to 2, which indicates that the graphene is a monolayer and the defect density is very small. With the temperature decrease in Zone III, the intensity of D peak increases, indicating the graphene quality decreased significantly. The reason should be that the diffusion rate of carbon atoms on the metal surface reduced at lower temperatures and more graphene crystal nuclei are formed, resulting in more grain boundaries and stacking [21]. When the temperature decreased to 200 °C, there are no graphene signals observed. This is because the temperature in Zone III is too low, causing carbon radicals to recombine before reaching the Cu surface. Figure 2b,c show the Raman intensity ratio mapping of *I*_2*D*_/*I_G_* and *I_D_*/*I_G_* growth at Zone III = 300 °C. The mean intensities of *I*_2*D*_/*I_G_* and *I_D_*/*I_G_* are 1.7 and 0.1, respectively, which means that the obtained graphene is mostly monolayer and of high quality. The FWHM (2D) also is a good argument in a discussion about the number of layers. We randomly selected 10 points in the graphene mapping results and the calculated average FWHM (2D) is 35 cm^−1^. This indicates that the whole graphene film is mostly monolayer. In order to further verify the effect of gradient temperature on the pyrolysis of methane and growth of graphene, we kept the temperature of the three zones consistent to conduct a comparative experiment of graphene growth. Figure 2d shows the Raman spectra of the graphene grown without gradient temperature. At 300 °C, the graphene quality decreased significantly compared with the graphene shown in Figure 2a. As the growth temperature increased, we found that although the graphene quality began to improve, to obtain similar quality to the graphene grown at 300 °C with gradient temperature, the growth temperature needs to be at least 600 °C. We attribute this phenomenon to the fact that, when Zone I has a high temperature, the carbon free radicals can still retain a relative high activation energy and diffusion rate when they move to the substrate surface with a low temperature (Zone III). Figure 2e shows the transmission spectrum of the graphene. The transmittance is about 97% in the wavelength range of 450 nm to 1000 nm. Especially around 680 nm, the transmittance is 97.7%, which is completely consistent with the theoretical value of monolayer graphene [22]. Transmission electron microscopy (TEM) images and corresponding selected area electron diffraction (SAED) patterns (Figure 2f) further confirm that the graphene is monolayer.

**Figure 3:**
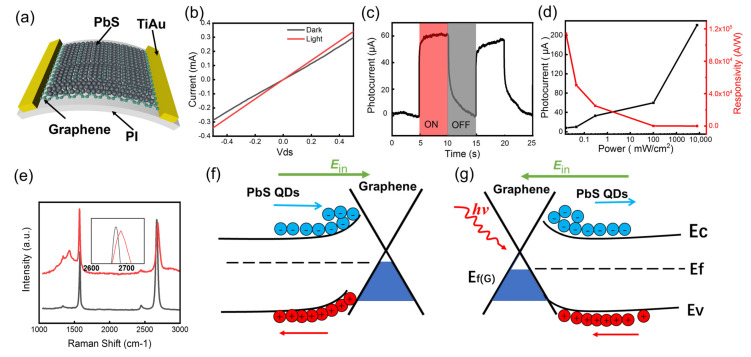
(**a**) Schematic diagram of the graphene–PbS photodetector. (**b**) The I–V curve of the photodetector with or without laser irradiation. (**c**) Dynamic photocurrent output characteristics of the photodetector. (**d**) The responsivity and photocurrent versus the incident laser power intensity with a bias of 0.5 V. The red curve shows the relationship between the responsivity and the laser power intensity. The black curve shows the relationship between the photocurrent and the laser power intensity. (**e**) Raman spectra of the graphene before (black curve) and after (red curve) spin-coating PbS QDs. (**f**,**g**) Energy band diagram of the PbS–graphene heterojunction in dark (**f**) and under laser illumination (**g**).

Based on this low-temperature growth technique, we grew graphene film in situ on PI substrate and fabricated a graphene-based flexible photodetector. In order to make up the defect of weak light absorption of graphene, a layer of PbS QDs film was spin-coated on the graphene as light absorption layer. We obtained devices with quantum dots films of different thicknesses by changing the quantum dot concentration and the number of spinnings for several times. When PbS thickness is less than 80 nm, the device photocurrent increases with the increase of PbS film thickness, but when the thickness exceeds 80 nm, the device photocurrent begins to decrease. When the thickness of PbS is small (<80 nm), the increase of PbS thickness is beneficial to increase the light absorption of the PbS film, thereby improving the responsivity. However, when the thickness continues to increase (>80 nm), the incident light will be mainly absorbed by the PbS QDs close to the surface, and this part of the QDs is far away from the graphene, and the photogenerated holes cannot be effectively transferred to the graphene channel to generate effective photocurrent. Figure 3a shows the schematic diagram of the device. The device is a two-terminal device, and we only added a source-drain bias of 0.5 V during the measurement. The graphene channel has an area of 7 μm × 63 μm. PbS QDs have high quantum efficiency and can enhance the responsivity of the detector within their light absorption range. Figure 3b shows the I–V curves of the photodetector with and without 792 nm laser irradiation. We can see that the device has a significant photocurrent response. Figure 3c is the photocurrent of the devices within two optical switching cycles. The laser intensity we used is around 95 mW/cm^2^, therefore the device has a responsivity of about 131 A/W. Figure 3d shows the photocurrent and responsivity of the device versus the incident laser power density. The device responsivity increases as incident light intensity decreases. When the incident light power is 16 μW/cm^2^, the responsivity can even be larger than 1 × 10^5^ A/W (Supplementary Figure S1). However, a small laser intensity will also lead to a significant increase in the influence of noise. It is noteworthy that when the optical power density reaches 10,000 mW/cm^2^, due to the small area (7 μm × 63 μm) of the device, the actual light energy received by the device is not very large. At the same time, the substrate is a transparent material, so the heat generated on the device during the measurement is not large and will not cause damage to the device. 

The presence of PbS QDs can cause doping of graphene and change its electrical properties. Figure 3e shows the Raman spectra of graphene before (black curve) and after (red curve) spin-coating PbS QDs. We can see that the position of the 2D peak shifts to the right and the intensity of G peak increases after spin-coating PbS QDs, which indicates the concentration of electrons in graphene increases and obvious n-type doping [23,24,25]. The transfer curve of the device with the same structure on SiO_2_/Si substrate shows that the Dirac point of graphene moves from +25 V to 0 V (Supplementary Figure S2). The reason for this phenomenon is that a built-in electric field forms between graphene and the PbS QDs (Figure 3f), therefore, the electrons in PbS QDs flow to the graphene and the holes flow to the PbS. When light was irradiated on the device, as shown in Figure 3g, a large number of photogenerated electrons and holes will appear in the PbS QDs film. Under the action of a built-in electric field, the electron–hole pairs will be separated. The photogenerated holes will flow to the graphene, resulting in an increase of the concentration of holes and conductivity of the graphene. Therefore, a positive photocurrent will be generated. In addition, the built-in electric field hinders the recombination of photogenerated carriers, thereby prolonging the lifetime of these carriers. The photocurrent of photoconductive detectors is proportional to the lifetime of photogenerated carriers, so our device can have a high responsivity. The long lifetime of photogenerated carriers is also reflected in Figure 3c. It can be seen that, when the laser is off, the photocurrent has a slow disappearance speed. Compared with the devices without PbS QDs, can be improved by six orders of magnitude [26]. In Table 1, other graphene-based flexible detectors are compared with our work. It can be seen that our device has excellent performance. 

**Figure 4:**
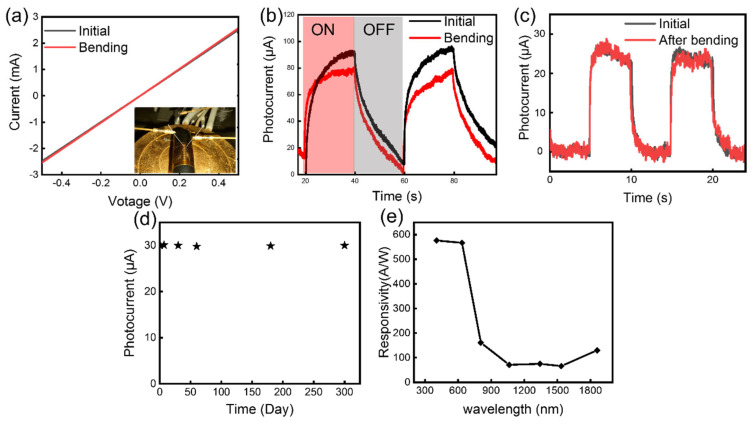
(**a**) The I–V curves of the photodetector with and without bending. (**b**) Dynamic photocurrent output characteristics of the photodetector with and without bending. (**c**) Photocurrent response of the device before (black curve) and after (red curve) a bending test for 100 times. (**d**) The photocurrent change of the device placed in the air within 300 days. (**e**) Responsivity of the device under different wavelengths (405 nm, 632 nm, 792 nm, 1064 nm, 1310 nm, 1550 nm, and 1860 nm). The laser power we used is around 0.4 μW.

We measured the performance of the device under bending condition. In order to effectively and stably control the bending of the device, we adhered the PI substrate to a cylinder. Figure 4a shows the I–V curves of the device with and without bending. The insert in Figure 4a shows the digital image of the measurement process. The result shows that bending does not significantly affect the conductivity of the device. Figure 4b shows the response of the device in two optical switches. We can see that the device response is slightly reduced during bending. We attribute this phenomenon to the changes of energy band with graphene. During bending, the graphene is stretched, which may create a small band gap in its energy bands, resulting in a decrease in its conductivity [27,28]. Figure 4c shows the response of the device within two optical switching cycles before and after a bending test carried out for 100 bends (Supplementary Figure S4). We can find that there is no obvious degradation. This is mainly because the graphene grown in situ can form a good contact with the substrate. At the same time, the electrodes were fabricated before the graphene growth, thereby avoiding the damage to the graphene during the device process. The above two points ensure the high reliability of the device, which is very important for the application of graphene-based devices. We also measured the long-term performance shift of the device placed in air (Figure 4d). The result shows that the device has high stability, and the performance will not change significantly after being placed in the air for 300 days. At the same time, we measured the photoresponse of the device under different laser wavelengths. Figure 4e shows that the responsivity exceeds 60 AW^−1^ under 405 nm–1860 nm laser irradiation. The wide spectral response enables the device to be used in more scenarios. Beside responsivity, the specific detectivity D* is also an important parameter to characterize the detector. We calculated D* by the following functions:

$$D^{*}=\frac{V_{s}}{V_{n}}\sqrt{\frac{\Delta f}{S}}\frac{1}{P}$$

where *V_s_* is signal voltage and *V_n_* is noise voltage, *S* is the detector area, Δ*f* is the equivalent noise bandwidth, and *P* is the laser power density. The calculated D* of device is 1.5 × 10^10^ Jones at room temperature with 0.5 V bias under a light intensity of 0.3 mW/cm^2^ (792 nm).

## 3. Conclusions

In summary, a method for in situ growth of graphene on a flexible substrate and its application in flexible photodetectors are reported in this work. A single-crystal Cu foil was bonded onto the PI substrate as a sacrificial layer and high-quality graphene film was grown on the Cu surface at 300 °C by a gradient descent temperature-controlled CVD system. After removing the sacrificial layer, graphene can be obtained directly on the substrate. For photodetector application, a PbS QDs layer was spin-coated on the graphene surface to form a graphene–PbS heterojunction photodetector. Under the irradiation of 792 nm laser, the responsivity of the device can be larger than 10^5^ A/W at room temperature. The device has a broad spectral response from 405 nm–1860 nm with a responsivity greater than 60 A/W. In situ growth and device process optimization improve the reliability of the device, and the photoresponse of the device did not change after multiple bending.

## 4. Methods

### 4.1. Transfer-Free Growth of Graphene on PI Film

Firstly, we made a Ti–Au electrode array on the PI substrate by lithography, sputtering, and lift-off. There are two main reasons for preparing electrodes before graphene growth. When the Cu sacrificial layer was prepared, the Cu foil needed to be bonded onto the PI surface. It is difficult to achieve a strong bond between metal and organic materials, therefore, the metal electrodes can act as an adhesion layer to assist the bonding of the substrate and the Cu foil. On the other hand, there is no need to fabricate electrodes after graphene growth, avoiding the damage to graphene by electrode process. The Cu foil was attached onto the PI substrate at a pressure of 100 kg/cm^2^, while the stage was heated to 50 °C and maintained for 1 h. 

Then, a PI substrate with a single-crystal Cu sacrificial layer was placed into CVD system. The graphene growth equipment we used is a three-temperature-zone quartz tube furnace with a diameter of 54 mm. The length of each temperature zone is 320 mm. The interval between the zones is 4 cm. The temperature of each zone can be heated and monitored independently. Before heating up, the quartz tube is first rinsed with argon gas for 5 min. Then, the three zones were set to 1000 °C, 750 °C, and 300 °C, respectively. The heating was carried out under a hydrogen atmosphere, the rate was controlled at 20 °C/min, and the air pressure was controlled at 2000 Pa. When the temperature of the first temperature zone reached 1000 °C, the vacuum was reduced to 110 Pa and the CH_4_/H_2_ (1.5 sccm/9 sccm) gas mixture was introduced to grow graphene. The growth time was set to 60 min. Subsequently, the Cu sacrificial layer was removed by penetrating etching. The etchant used was a CuSO_4_/HCl solution (CuSO_4_:HCl:H_2_O = 10 g:50 mL:50 mL). Before etching, the graphene surface was spun on a PMMA (polymethyl methacrylate) layer as protective layer. During etching, the etchant will penetrate through the PMMA and graphene. After the Cu was etched, graphene and PMMA can fall on the PI surface to realize in situ growth. Finally, we removed the PMMA by acetone/ethanol rinse.

### 4.2. The Fabrication and Measurement of the Photodetector

After the growth, the graphene film was patterned by photolithography followed by oxygen plasma etching. The size of graphene channel is 7 μm × 63 μm. Then, PbS QDs dissolved in n-hexane with a density of 20 mg/mL were spin-coated on the surface of graphene, followed by dropwise addition of 0.3 mL TBAI methanol solution (10 mg/mL) and held for 30 s to realize ligand exchange. The electrical properties were measured by an Agilent B1500A source meter. The lasers with different wavelengths were used as light sources to measure the response of the photodetector. 

## Figures and Tables

**Table 1:** Comparison of the similar photodetectors.

Substrate	Structure	In Situ Growth of Graphene	Wavelength	Responsivity	Specific Detectivity	Ref.
Polyimide	Graphene–PbS QDs	Yes	405–1860 nm	10^5^ A/W (792 nm)	1.5 × 10^10^ cmHz^1/2^/W	This work
PET	Graphene–PbS QDs	NO	990 nm	10^7^ A/W	-	[29]
polyimide	Graphene/Ultra-thin Silicon	NO	365 nm	0.47 A/W	2.5 × 10^10^ cmHz^1/2^/W	[30]
PEN	Graphene–copper phosphide QDs	NO	400–1550 nm	10^5^ A/W	5.9 × 10^12^ cmHz^1/2^/W	[31]
PET	Perovskite/graphene	NO	633 nm	10^7^ A/W	-	[32]
PET, PEN and PI	Graphene–semiconducting QDs	NO	300 to 2000 nm	10^5^ A/W	>3.7 × 10^11^ cmHz^1/2^/W	[33]
Poly (vinyl alcohol)	Graphene–chlorophyll a	NO	656 nm	200 A/W	-	[34]

## Data Availability

The data presented in this study are available on request from the corresponding author.

## Supplementary Materials

The following supporting information can be downloaded at: https://www.mdpi.com/article/10.3390/nano13081339/s1, Figure S1: Raman spectra of the graphene on PI (red curve) and SiO_2_/Si (black curve); Figure S2: Dynamic response of the device under 792 nm laser irradiation; Figure S3: Raman spectra of the graphene with pristine graphene (black), PbS-Graphene in dark (blue) and PbS-Graphene in light (red); Figure S4: PI substrate bending diagram.
