# Supplementary material for: In Situ Growth of Graphene on Polyimide for High-Responsivity Flexible PbS–Graphene Photodetectors
Source: Nanomaterials (Basel). 2023 Apr 12;13(8):1339. doi: 10.3390/nano13081339 (PMC10147023; doi:10.3390/nano13081339)
Supplement: Supplementary file 1 [file nanomaterials-13-01339-s001.zip › nanomaterials-2279540-supplementary.pdf]

Supplementary Materials for

In Situ Growth of Graphene on Polyimide for High–Responsivity Flexible PbS–Graphene Photodetectors

Liangchen Hu [1,†], Jun Deng [1,†], Yiyang Xie [1,*], Fengsong Qian [1], Yibo Dong [2,*], Chen Xu [1,*]

1 Key Laboratory of Optoelectronics Technology, Beijing University of Technology, Ministry of Education, Beijing 100124, China
2 Institute of Photonic Chips, University of Shanghai for Science and Technology, Shanghai 200093, China
* Correspondence: xieyiyang@bjut.edu.cn (Y.X.); dyb@usst.edu.cn (Y.D.); xuchen58@bjut.edu.cn (C.X.)
† These authors contributed equally to this work.

1. Raman spectra of graphene on different substrates

Raman spectra is an important method for characterizing graphene, but the peak of signals will lose in the noise in some substrates. As shown in the figure S1, the characteristic peaks of graphene cannot be clearly highlighted on the Polyimide surface, so we transferred the graphene grown on the Polyimide surface to the conventional SiO_2/Si surface for Raman spectra test.

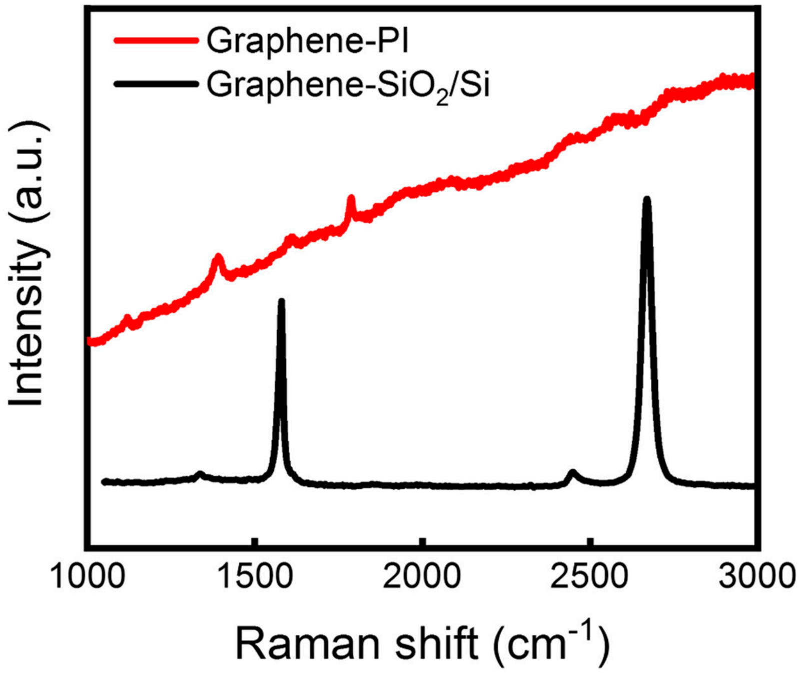

Supplementary Figure S1. Raman spectra of the graphene on PI (red curve) and SiO_2/Si (black curve).

2. **The response of photodetector under laser irradiation**

Figure S2 shows a device dynamic response at 792 laser irradiation (10 μW/cm^2). The device size is 10 μm ×70 μm, and a calculated device responsivity of approximately 1×10^5A/W.

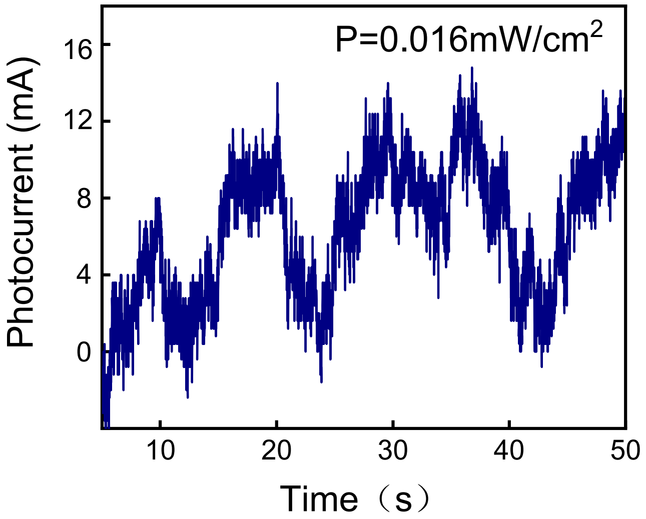

Supplementary Figure S2. Dynamic response of the device under 792nm laser irradiation.

3. Transfer characteristic curves of graphene FETs

Figure S3 show the transfer characteristics of graphene FETs (Field Effect Transistors) on SiO_2/Si substrate (channel size: 7 μm × 63 μm). After spinning PbS QDs, the graphene Dirac point moved from +25 V to near 0 V, and the graphene changed from P-type doping to near-intrinsic state, indicating that the charge exchange between graphene and QDs is exist. Under light irradiation, the graphene Dirac point shifted to the right, resulting the significantly changes photocurrent of the device.

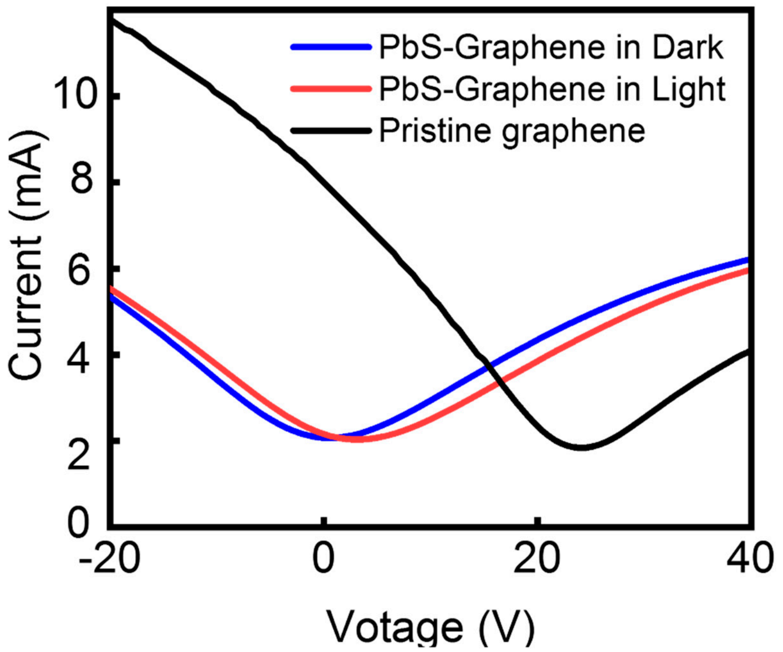

Supplementary Figure S3. Raman spectra of the graphene with pristine graphene (black), PbS-Graphene in dark (blue) and PbS-Graphene in light (red).

4. PI substrate bending mode

The substrate was bended more than 90° as shown in figure S4 and recover, repeat 100 times. Since it is a manual random bending angle, there is no fixed bending value.

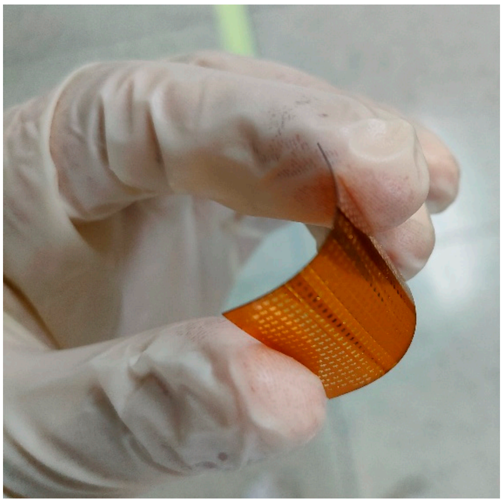

Supplementary Figure S4. PI substrate bending diagram
